# Global impact of anthropogenic NH_3_ emissions on upper tropospheric aerosol formation

**DOI:** 10.1073/pnas.2506658122

**Published:** 2025-10-27

**Authors:** Christos Xenofontos, Matthias Kohl, Samuel Ruhl, João Almeida, Lucía Caudillo-Plath, Romulo Cruz-Simbron, Lubna Dada, Jonathan Duplissy, Sebastian Ehrhart, Henning Finkenzeller, Kristina Höhler, Weimeng Kong, Felix Kunkler, Clara J. Lietzke, Bernhard Mentler, Aleksandra Morawiec, Antti Onnela, Pedro Rato, Birte Rörup, Douglas M. Russell, Meredith Schervish, Wiebke Scholz, Milin Kaniyodical Sebastian, Mario Simon, Eva Sommer, Yandong Tong, Nsikanabasi Silas Umo, Gabriela R. Unfer, Lejish Vettikkat, Boxing Yang, Wenjuan Yu, Imad Zgheib, Zhensen Zheng, Joachim Curtius, Neil M. Donahue, Richard C. Flagan, Hamish Gordon, Imad El Haddad, Armin Hansel, Hartwig Harder, Xu-Cheng He, Jasper Kirkby, Markku Kulmala, Katrianne Lehtipalo, Ottmar Möhler, Tuukka Petäjä, Mira L. Pöhlker, Siegfried Schobesberger, Dominik Stolzenburg, Mingyi Wang, Paul M. Winkler, Douglas R. Worsnop, Michael Höpfner, Rainer Volkamer, Andrea Pozzer, Jos Lelieveld, Theodoros Christoudias

**Affiliations:** ^a^Climate and Atmosphere Research Center, The Cyprus Institute, Nicosia 1645, Cyprus; ^b^Department of Atmospheric Chemistry, Max Planck Institute for Chemistry, Mainz 55128, Germany; ^c^The European Organization for Nuclear Research (CERN), Geneva 1211, Switzerland; ^d^Institute for Atmospheric and Environmental Sciences, Goethe University Frankfurt, Frankfurt am Main 60438, Germany; ^e^Cooperative Institute for Research in Environmental Sciences, University of Colorado Boulder, Boulder, CO 80309; ^f^Laboratory of Atmospheric Chemistry, Paul Scherrer Institute, Villigen 5232, Switzerland; ^g^Institute for Atmospheric and Earth System Research/Physics, Faculty of Science, University of Helsinki, Helsinki 00014, Finland; ^h^Institute of Meteorology and Climate Research Atmospheric Aerosol Research, Karlsruhe Institute of Technology, Karlsruhe 76021, Germany; ^i^Division of Chemistry and Chemical Engineering, California Institute of Technology, Pasadena, CA 91125; ^j^Institute for Ion Physics and Applied Physics, University of Innsbruck, Innsbruck 6020, Austria; ^k^Faculty of Physics, University of Vienna, Vienna 1090, Austria; ^l^Irvine Department of Chemistry, University of California, Irvine, CA 92697; ^m^Atmospheric Microphysics Department, Leibniz Institute for Tropospheric Research, Leipzig 04318, Germany; ^n^Department of Technical Physics, University of Eastern Finland, Kuopio 70211, Finland; ^o^TOFWERK, Thun 3645, Switzerland; ^p^Ionicon Analytik GmbH, Innsbruck 6020, Austria; ^q^Department of Chemical Engineering/Center for Atmospheric Particle Studies, Carnegie Mellon University, Pittsburgh, PA 15213; ^r^Department of Engineering and Public Policy, Carnegie Mellon University, Pittsburgh, PA 15213; ^s^Department of Chemistry, Carnegie Mellon University, Pittsburgh, PA 15213; ^t^Yusuf Hamied Department of Chemistry, University of Cambridge, Cambridge CB2 1EW, United Kingdom; ^u^School of Atmospheric Sciences, Joint International Research Laboratory of Atmospheric and Earth System Sciences, Nanjing University, Nanjing 210023, China; ^v^Aerosol and Haze Laboratory, Beijing Advanced Innovation Center for Soft Matter Science and Engineering, Beijing University of Chemical Technology, Beijing 100029, China; ^w^Finnish Meteorological Institute, Helsinki 00101, Finland; ^x^Institute for Materials Chemistry, Technische Universität (TU) Wien, Vienna 1060, Austria; ^y^Department of the Geophysical Sciences, The University of Chicago, Chicago, IL 60637; ^z^Aerodyne Research Inc., Billerica, MA 01821; ^aa^Institute of Meteorology and Climate Research Atmospheric Trace Gases and Remote Sensing, Karlsruhe Institute of Technology, Karlsruhe 76021, Germany

**Keywords:** new particle formation, anthropogenic NH_3_ emissions, UTLS, CCN, AOD

## Abstract

Anthropogenic ammonia (NH_3_) emissions have significantly increased in recent decades due to enhanced agricultural activities, contributing to global air pollution. While the effects of NH_3_ on surface air quality are well documented, its influence on particle dynamics in the upper troposphere-lower stratosphere (UTLS) and related aerosol impacts remain unquantified. NH_3_ reaches the UTLS through convective transport and can enhance new particle formation (NPF). This modeling study evaluates the global impact of anthropogenic NH_3_ on UTLS particle formation and quantifies its effects on aerosol loading and cloud condensation nuclei (CCN) abundance. We use the EMAC Earth system model, incorporating multicomponent NPF parameterizations from the CERN CLOUD experiment. Our simulations reveal that convective transport increases NH_3_-driven NPF in the UTLS by one to three orders of magnitude compared to a baseline scenario without anthropogenic NH_3_, causing a doubling of aerosol numbers over high-emission regions. These aerosol changes induce a 2.5-fold increase in upper tropospheric CCN concentrations. Anthropogenic NH_3_ emissions increase the relative contribution of water-soluble inorganic ions to the UTLS aerosol optical depth (AOD) by 20% and increase total column AOD by up to 80%. In simulations without anthropogenic NH_3_, UTLS aerosol composition is dominated by sulfate and organic species, with a marked reduction in ammonium nitrate and aerosol water content. This results in a decline of aerosol mass concentration by up to 50%. These findings underscore the profound global influence of anthropogenic NH_3_ emissions on UTLS particle formation, AOD, and CCN production, with important implications for cloud formation and climate.

## Significance

Ammonia (NH_3_) emissions from human activities can significantly influence aerosol processes in the upper troposphere and lower stratosphere (UTLS). Using an Earth system model, we show that anthropogenic NH_3_ strongly enhances new particle formation and growth, leading to substantial changes in UTLS aerosol composition and abundance. These changes can enhance cloud condensation nuclei concentrations by a factor of 2.5 in the upper troposphere over high-emission regions. In addition, aerosol optical depth can increase by up to 80%, potentially affecting climate. Our findings underscore the need to account for UTLS NH_3_-driven aerosol processes in Earth system models to improve predictions of atmospheric composition and cloud effects in climate scenarios.

Ammonia (NH_3_) plays a critical role in atmospheric new particle formation (NPF) by stabilizing acid–base nucleation (1). It also contributes to particle growth through condensation (2, 3). The resulting particles can affect climate by scattering and absorbing solar radiation (4, 5), while they can also act as cloud condensation nuclei (CCN) that seed cloud droplets (6–8). It is estimated that approximately half of all CCN—and nearly all in the upper troposphere (UT)—originate from NPF (9, 10). The specific contribution of anthropogenic NH_3_ emissions to particle formation in the upper troposphere-lower stratosphere (UTLS), CCN production, and their influence on atmospheric aerosol remains unquantified. This study aims to address this knowledge gap.

NH_3_ is released into the atmosphere from biogenic and anthropogenic sources (11). While biogenic sources such as soil microbial activity (12), decomposition of animal waste (13), and vegetation (14) contribute to global NH_3_ emissions, anthropogenic activities remain the dominant source. Agricultural practices, including fertilizer application (15) and livestock farming (16), account for 80 to 90% of global NH_3_ emissions (17), with additional contributions from industrial processes (18), vehicle emissions (19), and biomass burning (20). These emissions are concentrated in major agricultural regions, primarily Europe (21), the United States (22), South and East Asia (23, 24), with Central Africa (25) and South America (26) also identified as high-emission regions. Previous studies indicated that NH_3_ emissions can increase CCN concentrations by up to 80% over the Asian monsoon region (27). However, the exact contribution of anthropogenic NH_3_ and its global impact on CCN was not addressed. As anthropogenic NH_3_ emissions are projected to double by 2100 (28), it is crucial to understand their global impact in terms of UTLS particle formation and climatic influence through CCN and aerosol optical depth (AOD) changes.

As most NH_3_ sources are situated at the Earth’s surface, only a small fraction can reach the UTLS due to the short atmospheric lifetime (29). This limited longevity results from interaction with aerosol and clouds, which promotes efficient removal through scavenging and condensation processes (30). Consequently, gas-phase NH_3_ concentrations can be expected to decrease sharply with altitude, resulting in low levels in the UTLS, particularly over marine regions. This is supported by various in situ observations (31, 32). However, due to the semivolatile nature, hydrophilicity, and highly variable ambient concentrations of NH_3_, in situ observations in the UTLS are subject to considerable uncertainty (33). Analysis of the MIPAS (Michelson Interferometer for Passive Atmospheric Sounding) spaceborne instrument infrared limb-emission spectra has revealed elevated concentrations of NH_3_ (reaching up to 30 pptv) in the UTLS over emission hotspots (34). Höpfner et al. (34) further noted that limited modeled outputs are available for comparison with these observations.

Accurately representing NH_3_-induced particle formation in the UTLS remains challenging with atmospheric models. This is partly because most of the models do not include the recently discovered (35) synergistic interaction of NH_3_ with nitric acid (HNO_3_) and sulfuric acid (H_2_SO_4_), which results in NPF, particularly in typical UTLS conditions. Furthermore, atmospheric models often do not accurately represent the dynamics of particle growth to CCN sizes, a critical step linking NPF processes to cloud formation and climate (7).

The contribution of NH_3_ to the formation of ammonium nitrate (NH_4_NO_3_) and its effects on AOD are another significant source of uncertainty in atmospheric models (36). This is primarily due to the variability and limitations in emission inventories (37, 38). It is suggested that NH_4_NO_3_ may become the largest contributor to anthropogenic AOD by the end of the 21st century due to increasing agricultural NH_3_ emissions (39). Aircraft observations have revealed high concentrations of NH_3_ as well as NH_4_NO_3_ aerosols in the UTLS (40, 41), where a low scavenging rate allows them to persist longer than in the lower troposphere (42). This extended residence time can enhance their contribution to AOD. Representing all these processes in atmospheric models is critical for accurately predicting UTLS particle formation processes, their contribution to atmospheric composition, and their broader implications for climate (43, 44).

This study quantifies the global influence of anthropogenic NH_3_ on UTLS particle formation and its implications for CCN concentrations and AOD. Uniquely, we evaluate the modeled UTLS NH_3_ precursor concentrations against MIPAS observations, and we take into account the interactions between NH_3_, HNO_3_, and H_2_SO_4_ in NPF. Recently published NPF parameterizations from the CERN CLOUD (Cosmics Leaving Outdoor Droplets) chamber (2, 35, 45–48) are implemented into the state-of-the-art EMAC (ECHAM/MESSy Atmospheric Chemistry) Earth system model (49). We note that the recently discovered UTLS organic nucleation (50) is not yet included in our study due to the lack of available parameterizations. Isoprene-derived oxidation products can significantly increase NPF in convective outflow regions over tropical rainforests (51). We quantify the contribution of convective updrafts to NPF rates and particle number concentrations in the UTLS. Furthermore, by comparing multiyear simulations with and without global anthropogenic NH_3_ emissions, we estimate the changes induced by anthropogenic NH_3_ in the UTLS aerosol composition, number, and mass concentrations. The relative importance of the various UTLS aerosol components to the total AOD is calculated, critical for simulating the aerosol direct climatic effect (52). Finally, we quantify the impact of anthropogenic NH_3_ emissions on UT CCN number concentrations, revealing notable changes that could influence cloud formation.

## Results and Discussion

### Convection-Induced NPF.

**Fig. 1.**
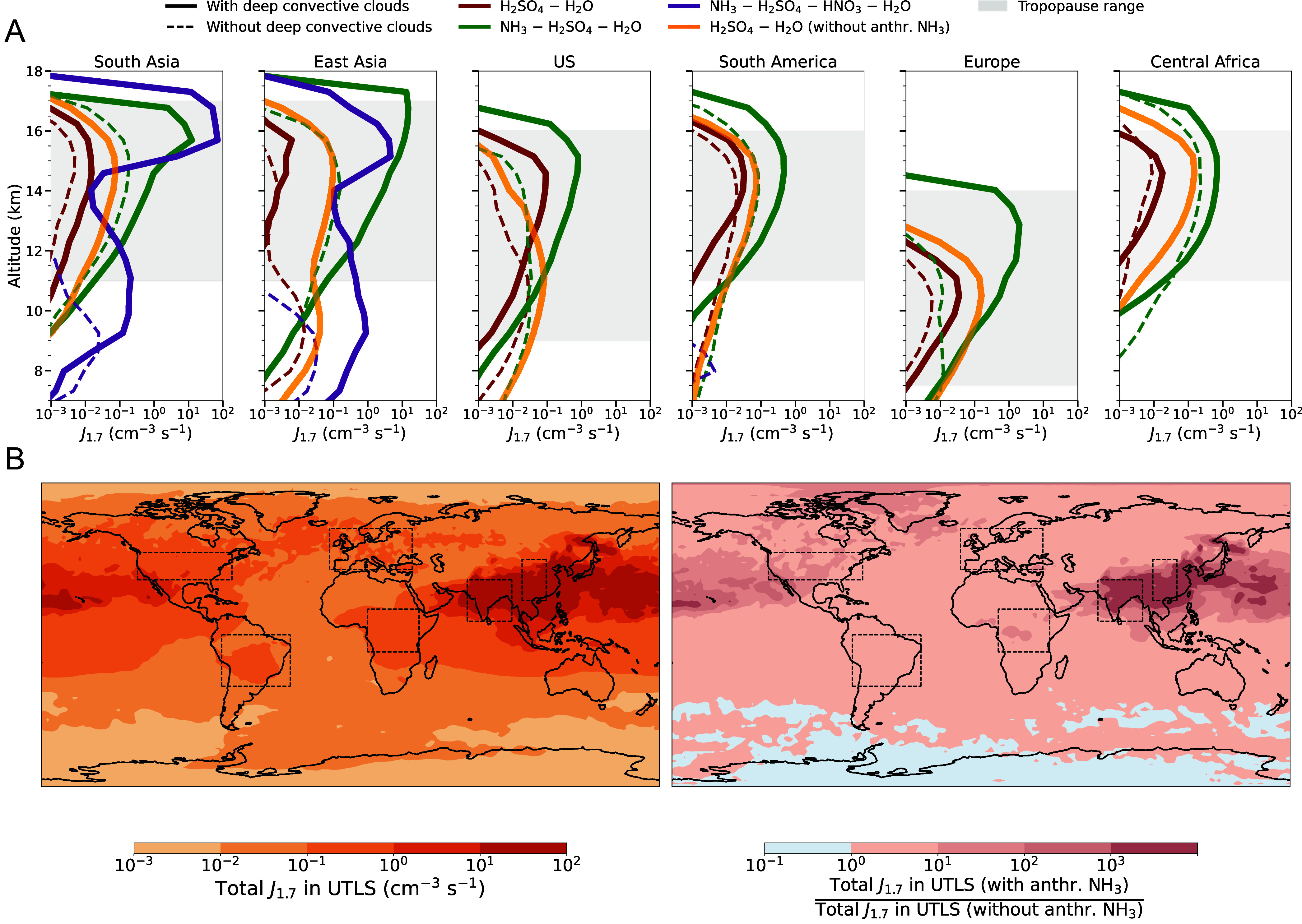
(*A*) Average simulated nucleation rates at 1.7 nm diameter ($J_{1.7}$), calculated at ambient temperature and pressure, as a function of altitude. The six major NH_3_ emission regions are indicated with dashed outlines over the map in (*B*): South and East Asia, the United States, Europe, South America, and Central Africa. The nucleation mechanisms included are synergistic NH_3_–H_2_SO_4_–HNO_3_–H_2_O (purple); ternary NH_3_–H_2_SO_4_–H_2_O (green); binary H_2_SO_4_–H_2_O without anthropogenic NH_3_ emissions (orange); and binary H_2_SO_4_–H_2_O in the presence of anthropogenic NH_3_ emissions (brown). The solid lines denote conditions with deep convective clouds, whereas the dashed lines represent instances of a quiescent atmosphere without convective clouds. In some regions, the purple lines representing synergistic nucleation are not visible because the contribution of this mechanism is very low, resulting in $J_{1.7}$ values below the range shown in the plot. In contrast, synergistic nucleation is enhanced in Asia. The gray shaded area represents the tropopause altitude range in each region. *(B)* Global map of total simulated UTLS $J_{1.7}$ with anthropogenic NH_3_ (*Left*) and the enhancement ratio due to NH_3_ (*Right*). The enhancement is defined as the ratio of UTLS *J*_1.7_ with anthropogenic NH_3_ to UTLS *J*_1.7_ without. Values greater than 1 indicate an increase in *J*_1.7_ due to NH_3_ (in red), while values below 1 indicate a reduction (in blue). The results are averaged over the entire period from 2003 to 2019.

Convection is thought to influence NPF by transporting precursor vapors from the boundary layer to higher altitudes in the UTLS (2). We investigate the impact of convection-induced NPF by simulating nucleation rates at 1.7 nm diameter (*J*_1.7_) in the UTLS, showing average global and regional profiles from 2003-2019 (Fig. 1). Here, the UTLS is defined as approximately 7 to 18 km altitude, encompassing the variable tropopause region. The simulated time period enables a direct comparison of NH_3_ concentrations with satellite data from MIPAS, IASI (Infrared Atmospheric Sounding Interferometer), and AIRS (Atmospheric Infrared Sounder). The model reproduces the observed NH_3_ concentrations with a normalized mean bias within ±15% (*Methods* and *SI Appendix*).

Fig. 1*A* shows the vertical profiles of *J*_1.7_ over the NH_3_ strong-emission regions. Composites of days with deep convective clouds are compared to clear-sky days for six major NH_3_ emission hotspot regions: South and East Asia, the United States (US), Europe, South America, and Central Africa. The results indicate that ternary ${\text{NH}}_{3}-{\text{H}}_{2}{\text{SO}}_{4}-{\text{H}}_{2}\text{O}$ nucleation is overall the dominant mechanism across all regions, with rates significantly enhanced in the presence of deep convective clouds. Binary ${\text{H}}_{2}{\text{SO}}_{4}-{\text{H}}_{2}\text{O}$ nucleation is, on average, two to three orders of magnitude lower than ternary nucleation. Synergistic ${\text{NH}}_{3}-{\text{H}}_{2}{\text{SO}}_{4}-{\text{HNO}}_{3}-{\text{H}}_{2}\text{O}$ nucleation is more prominent over South and East Asia. These regions are characterized by frequent convective updraft events, in particular during the Asian monsoon, substantial anthropogenic NH_3_ emissions and abundant nitrogen oxides (${\text{NO}}_{\text{x}}$), e.g., from lightning (53).

Zhao et al. (10) incorporated the NH_3_-enhanced NPF CLOUD parameterizations to estimate the contributions of different nucleation pathways to the total *J*_1.7_. In our results, the peak *J*_1.7_ is, on average, an order of magnitude higher than that reported by Zhao et al. over NH_3_ hotspots. This difference arises from distinct averaging approaches. Zhao et al. averaged over all days throughout their simulation period, whereas we separately average over composites of days with and without deep convection. This allows us to focus on and quantify the specific enhancement in *J*_1.7_ associated with deep convection (27). Under clear-sky conditions over NH_3_ hotspots, our peak *J*_1.7_ values are comparable to those in Zhao et al.

In our additional baseline simulation, we switch off anthropogenic NH_3_ emissions globally, with all other conditions kept constant. This ensures that any differences between the two simulations arise solely from the absence of anthropogenic NH_3_. In this scenario, the binary ${\text{H}}_{2}{\text{SO}}_{4}-{\text{H}}_{2}\text{O}$ nucleation mechanism dominates. However, on average, the peak *J*_1.7_ is one to three orders of magnitude lower than NH_3_-enhanced nucleation. The spatial distribution highlights that with anthropogenic NH_3_, there are strong enhancements in the total UTLS *J*_1.7_ over industrialized and agricultural regions, particularly South and East Asia and the United States, compared to the scenario without anthropogenic NH_3_ (Fig. 1*B*).

### Influence on UTLS Aerosol Composition and Abundance.

**Fig. 2.**
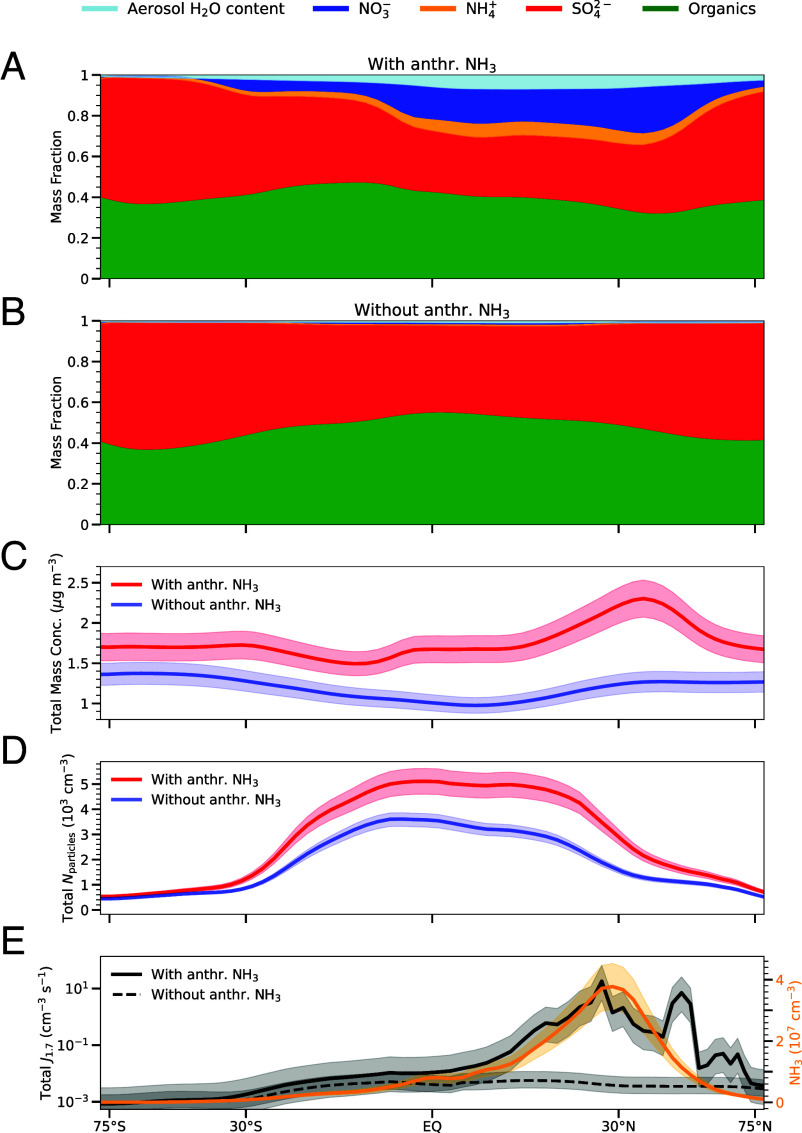
Simulated latitudinal profiles of UTLS aerosol components by mass (aerosol H_2_O content, NO_3_^–^, NH_4_^+^, SO_4_^2–^, and organics) (*A*) with, and (*B*) without anthropogenic NH_3_ emissions. Latitudinal profiles of simulated total particle (*C*) mass and (*D*) number concentrations ($N_{\text{particles}}$) in the UTLS with (red line) and without (blue line) anthropogenic NH_3_. Particle size ranges from 2 nm to 1,000 nm. (*E*) Simulated latitudinal profiles of the total UTLS nucleation rate at 1.7 nm diameter (*J*_1.7_) with (solid black line) and without (dashed black line) anthropogenic NH_3_. The UTLS concentrations of anthropogenic NH_3_ are shown in orange. All concentrations and *J*_1.7_ are calculated at ambient temperature and pressure. The lines and the shaded areas represent the mean and the interannual variability (temporal SD) for the corresponding latitude, respectively. Modeled outputs are averaged within the 10 to 15 km altitude range over the entire period from 2003 to 2019.

Understanding the impact of anthropogenic NH_3_ on aerosol composition and abundance in the UTLS is important for assessing its influence on climate (34, 40). The coincidence of latitudinal profiles of NH_3_ concentration, total *J*_1.7_, aerosol mass fraction, total aerosol mass, and number concentrations ($N_{\text{particles}}$) in the UTLS can be seen in Fig. 2.

The influence of anthropogenic NH_3_ emissions on the average mass fractions of key aerosol components-sulfate (${\text{SO}}_{4}^{2-}$), nitrate (${\text{NO}}_{3}^{-}$), ammonium (${\text{NH}}_{4}^{+}$), water (${\text{H}}_{2}$O), and organics is shown in Fig. 2 *A* and *B*. Over emission hotspots, NH_3_ markedly increases ${\text{NO}}_{3}^{-}$ and ${\text{NH}}_{4}^{+}$ aerosol mass fractions in the UTLS, while its absence drastically reduces ${\text{NO}}_{3}^{-}$ and nearly eliminates ${\text{NH}}_{4}^{+}$ and aerosol ${\text{H}}_{2}$O content. ${\text{NH}}_{4}{\text{NO}}_{3}$ evaporates at the higher temperatures of the lower troposphere but remains entirely in the particulate phase in the UTLS (40). Below roughly 233 K, it is essentially impossible for NH_3_ and HNO_3_ to coexist in the gas phase, and the formation of ${\text{NH}}_{4}{\text{NO}}_{3}$ is practically irreversible (54). We find that, on average, organics constitute 40% of the simulated UTLS aerosol mass. Secondary organic aerosols from volatile organic compound oxidation account for 90% of this organic mass, while primary organic aerosols from biomass and fossil fuel combustion account for the remaining 10%.

As shown in Fig. 2*C*, the total simulated aerosol mass concentration is increased in the UTLS when anthropogenic NH_3_ is present, with the largest increase observed from 0 to 40°N. This increase in mass concentration due to ${\text{NH}}_{4}{\text{NO}}_{3}$ coincides with the increase in NH_3_ concentration. In addition, $N_{\text{particles}}$ (Fig. 2*D*) are increased by up to 2,000 cm^−3^ between 30°S and 30°N with anthropogenic NH_3_ relative to the scenario without anthropogenic NH_3_ emissions. The presence of NH_3_ enhances *J*_1.7_ (Fig. 2*E*) in the tropics and midlatitudes by up to three orders of magnitude. The alignment of the peak *J*_1.7_ and aerosol mass concentrations with high NH_3_ concentration is evidence of its importance in both NPF and the growth of particles in the UTLS.

### Effects on AOD.

**Fig. 3.**
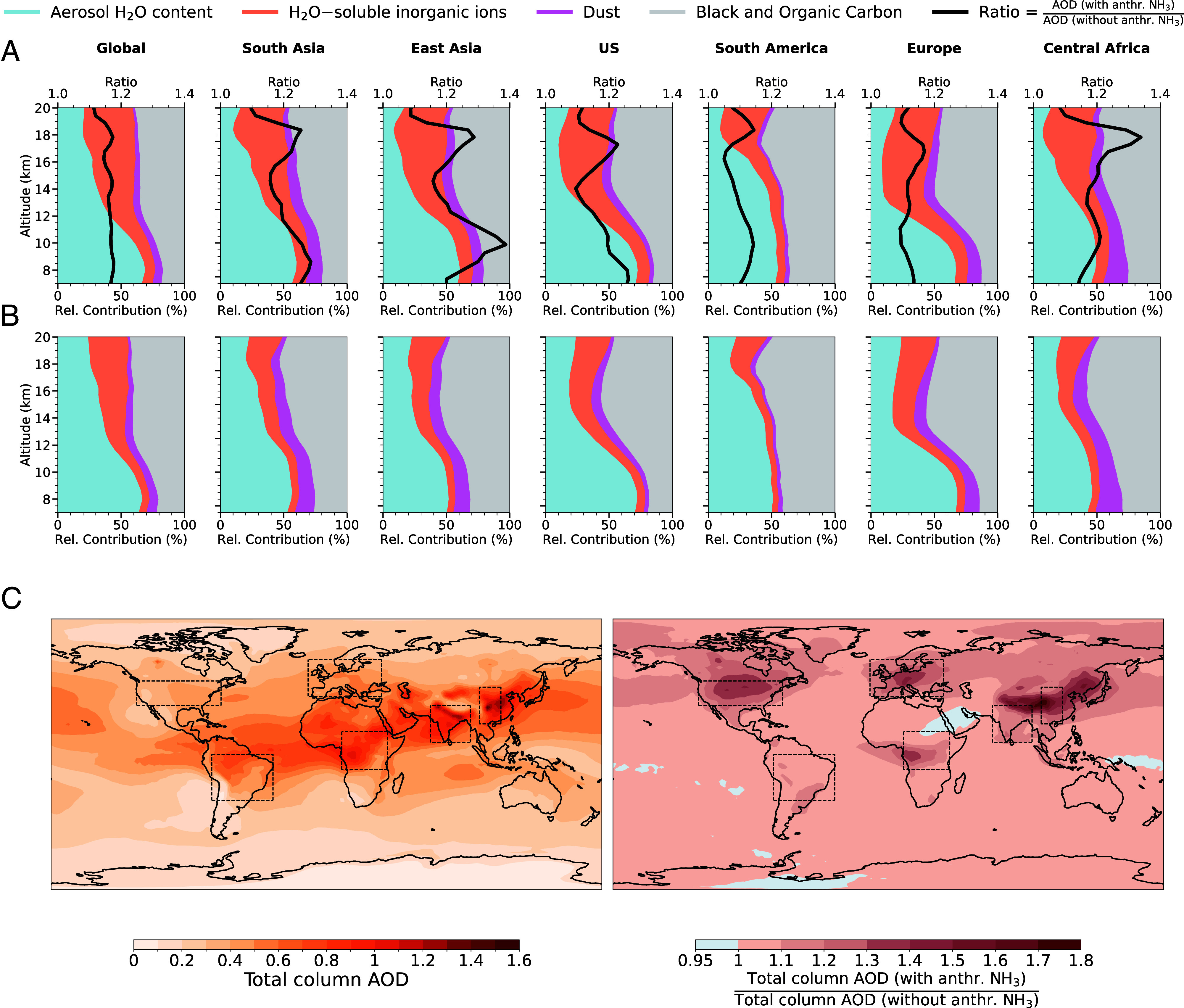
Vertical distribution of the contribution of different UTLS AOD components to the total AOD over NH_3_ emission hotspots (*A*) with and (*B*) without anthropogenic NH_3_. The relative contributions of aerosol components (H_2_O, H_2_O-soluble inorganic ions, dust, black and organic carbon) to the total AOD (550 nm) are shown as stacked colored regions. The black line represents the ratio of AOD with anthropogenic NH_3_ to AOD without, highlighting the relative enhancement due to NH_3_ emissions at each altitude. The tropopause altitude range for each region is specified in Fig. 1*A*. (*C*) Total atmospheric column AOD at 550 nm in the presence of anthropogenic NH_3_ (*Left*). AOD total column enhancement ratio is defined as the ratio of total column AOD with anthropogenic NH_3_ to without (*Right*). Values greater than 1 indicate an increase in AOD due to NH_3_ (in red), while values below 1 indicate a decrease (in blue). NH_3_ emission hotspots are marked on the map. The results are calculated at ambient conditions and averaged over the entire period from 2003 to 2019.

The influence of anthropogenic NH_3_ emissions on the total AOD at 550 nm and the different UTLS aerosol components of the AOD for each case is presented in Fig. 3. The individual aerosol components considered include black and organic carbon, dust, H_2_O-soluble inorganic ions, and the aerosol H_2_O content.

In Fig. 3 *A* and *B*, we compare the changes in the UTLS AOD components in the presence and absence of anthropogenic NH_3_, respectively. The contribution of the H_2_O-soluble inorganic ions to the total UTLS AOD (550 nm) increases and reaches an average 40 to 50% in the presence of anthropogenic NH_3_. This increase is especially pronounced over regions with intense agricultural and industrial activities, such as South and East Asia. The peaks in the AOD enhancement ratio due to anthropogenic NH_3_ align with enhanced contributions from H_2_O-soluble inorganic ions. On average, this peak enhancement ratio varies from 20 to 40% over high-emission regions. As a strong base, NH_3_ facilitates the partitioning of HNO_3_ and H_2_O into the aerosol phase, leading to increased formation of H_2_O-soluble compounds (such as ${\text{NH}}_{4}{\text{NO}}_{3}$), which in turn enhances AOD (55). In the absence of anthropogenic NH_3_, the contribution of the H_2_O-soluble inorganic ions to the total AOD is reduced on average by 20% in the UTLS.

The total atmospheric column AOD (from the surface to the top of the atmosphere) at 550 nm exhibits a global increase in response to anthropogenic NH_3_ emissions (Fig. 3*C*). The strongest enhancements of AOD are observed over major NH_3_ source regions. The increase in AOD can reach up to 60 to 80% in the presence of anthropogenic NH_3_ in these regions compared to its absence. This enhancement results from NH_3_-driven aerosol formation in the UTLS and lower altitudes, indicating a significant influence on aerosol optical properties with potential implications for atmospheric radiative transfer and climate.

### Global Impact of Anthropogenic NH_3_ on UT CCN.

We model the global impact of anthropogenic NH_3_ emissions on CCN concentrations at 0.4% supersaturation (CCN_0.4%_) in the UT. Our simulations show that particle number concentrations ($N_{\text{particles}}$) in the UTLS (Fig. 2*D*) exhibit a strong response to anthropogenic NH_3_ emissions, especially in the Northern Hemisphere, where these emissions are most concentrated. This increase in $N_{\text{particles}}$ can contribute to the total CCN_0.4%_ in the UT, with potential implications for cloud formation (7). As these particles continue to grow, they may be transported downward with descending air masses. Upon reaching lower altitudes, they can further contribute to cloud formation in the lower troposphere (7).

**Fig. 4.**
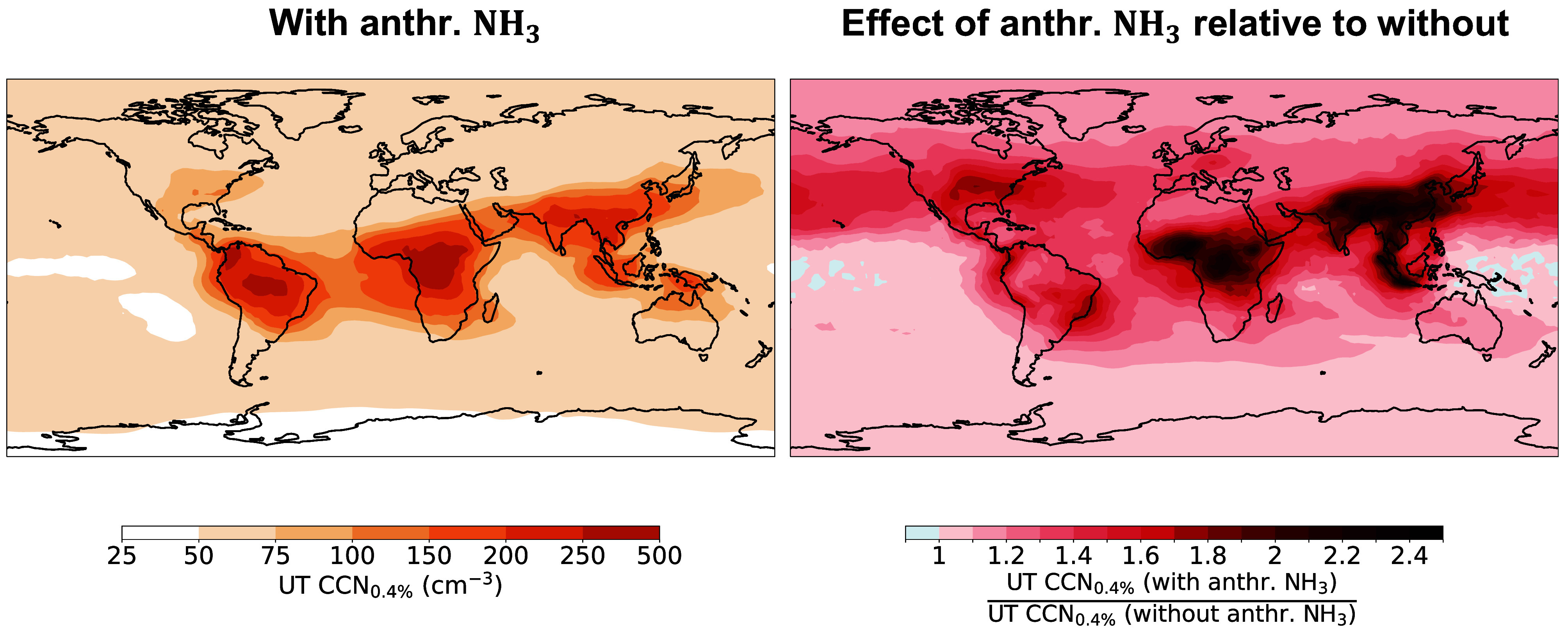
Global maps of cloud condensation nuclei concentrations at 0.4% supersaturation (CCN_0.4%_) in the UT, averaged within the 10 to 13 km altitude range. The *Left* panel illustrates the absolute UT CCN_0.4%_ values in the presence of anthropogenic NH_3_. The *Right* panel shows the enhancement ratio, defined as the ratio of UT CCN_0.4%_ values with anthropogenic NH_3_ to those without. Values greater than 1 indicate an increase due to NH_3_ (in red), while values below 1 indicate a reduction (in blue). The results are calculated at ambient conditions and averaged over the entire period from 2003 to 2019.

In the UT, the CCN_0.4%_ concentration is substantially enhanced by anthropogenic NH_3_ (Fig. 4*B*). This enhancement results from the doubling of UTLS aerosol abundance and is most pronounced over regions with strong agricultural and industrial emissions, where CCN_0.4%_ concentrations exceed 200 cm^−3^—up to a 2.5-fold increase. In addition, over the oceans, downwind of NH_3_ emissions and in the absence of local anthropogenic sources, CCN_0.4%_ concentrations increase substantially. A significant portion of cirrus ice crystals can originate from cloud droplets that first condense onto CCN before freezing (56–58). Changes in CCN concentrations at the altitudes of convective anvils, contrails, or cirrus clouds influence ice crystal formation and concentrations (56, 59) that can significantly affect climate by altering the radiative properties of cirrus clouds (60, 61).

### Sensitivity Analysis.

Anthropogenic emissions in our simulations are derived from the Community Emissions Data System (CEDS) (62). Uncertainties in CEDS arise from inaccuracies in activity data (e.g., energy consumption), incomplete or outdated emission factors, and limited information on the enforcement of control technologies, particularly in emerging economies. These uncertainties vary across different compounds, sectors, regions, and time periods. For NH_3_, the main source of uncertainty is the variability in agricultural practices and emission reporting.

To assess the sensitivity of our results to this uncertainty, we performed additional simulations in which global NH_3_ emissions were perturbed by ±15%. This range was based on the global normalized mean bias (NMB) between EMAC-simulated and IASI-retrieved NH_3_ concentrations, which is approximately 15% (*Methods* and *SI Appendix*). We find that perturbing global NH_3_ emissions by ±15% leads to regional variations in UT *J*_1.7_, CCN_0.4%_, and AOD within 20% in spatial distributions and vertical profiles (*SI Appendix*, Figs. S1–S3).

In South and East Asia, the NMB is 27% and 50%, respectively, and up to 200% at certain altitudes. To quantify the influence of this uncertainty on our results, we carried out region- and season-specific perturbation sensitivity simulations in which NH_3_ emissions were scaled based on the EMAC-to-IASI ratio in each hotspot region for each month. During the summer monsoon season in South and East Asia, variations in UT *J*_1.7_, CCN_0.4%_, and AOD due to NH_3_ emission perturbations can reach up to 30%. Elevated uncertainties are also observed during spring, while winter and autumn show minimal sensitivity across all hotspot regions (*SI Appendix*, Figs. S1 and S2). Since AIRS shows better agreement with EMAC than IASI, these seasonal and regional uncertainties should be regarded as conservative estimates, and the actual uncertainties may be smaller.

To examine the sensitivity of UTLS particle formation to long-term emission trends, we analyzed time series of anthropogenic emissions (from CEDS) and total UTLS *J*_1.7_ over NH_3_ hotspots (*SI Appendix*, Fig. S4). Individual species emissions in certain regions exhibit temporal trends, with increasing NH_3_ fluxes in South Asia, and declining SO_2_ and ${\text{NO}}_{\text{x}}$ in Europe and the United States due to emission controls. The response to these trends of *J*_1.7_ is influenced by a complex interplay of the subsequent reactions producing multiple nucleation precursors (e.g. oxidation of ${\text{SO}}_{2}$ to ${\text{H}}_{2}{\text{SO}}_{4}$), the presence of natural emissions (such as lightning ${\text{NO}}_{\text{x}}$), and transport dynamics, in particular convective updrafts. The variability in *J*_1.7_ is thus not solely modulated by the trend of individual species emissions over each region, and without a consistent long-term trend across all regions. The presence of deep convective clouds substantially enhances *J*_1.7_ values across all regions; thus it is expected that, other than emission trends, meteorological factors such as monsoon duration and intensity, play an important role in year-to-year temporal variability.

## Conclusions

This study underscores the critical role of anthropogenic NH_3_ emissions in driving particle formation in the UTLS. By incorporating recently published NPF parameterizations derived from the CERN CLOUD experiment into the EMAC model, we quantified the impact of anthropogenic NH_3_ emissions on UTLS particle formation by comparing a simulation with anthropogenic NH_3_ emissions to a baseline simulation without anthropogenic NH_3_.

Our simulations show that convective transport of anthropogenic NH_3_ enhances UTLS NPF rates by one to three orders of magnitude over NH_3_ hotspots. This NH_3_-driven particle formation and growth leads to a doubling of UTLS particle numbers over emission hotspots compared to a scenario without anthropogenic NH_3_. These particles can contribute to a 2.5-fold enhancement of UT CCN concentrations over high-emission regions, and smaller but significant changes over the oceans. Eliminating anthropogenic NH_3_ would nearly deplete the UTLS aerosol ${\text{NH}}_{4}^{+}$ and H_2_O content, and substantially reduce ${\text{NO}}_{3}^{-}$ levels. These chemical composition changes lead to a global reduction in the total UTLS aerosol mass concentration, reaching up to 50% over Asia. This significant decrease in global aerosol loading also translates to a lower AOD without NH_3_, up to 80% in the Northern Hemisphere and up to 10% in the Southern Hemisphere. These findings highlight the role of anthropogenic NH_3_ in affecting UTLS aerosol abundance and composition, cloud formation, and aerosol optical properties, with potential implications for climate.

Our study opens future research directions by highlighting the need to integrate UTLS NH_3_-driven aerosol formation into Earth system models to improve the predictive accuracy of atmospheric composition and cloud effects in climate scenarios. While this study focuses on global CCN and AOD, further research should quantify the radiative forcing of UTLS NH_3_-driven aerosol changes. In addition, anthropogenic NH_3_ may influence ice-nucleating particle activity, with potential consequences for cloud glaciation and mixed-phase cloud processes (63). A deeper understanding of these processes is essential for assessing the role of NH_3_ in aerosol–cloud–climate interactions. In particular, more targeted observations and improved emission estimates over South and East Asia during the summer monsoon season are needed to better constrain the role of deep convection in NH_3_ transport and UTLS aerosol formation.

Current policies primarily target NH_3_ emissions to address air quality concerns (64), with recent directives encouraging additional NH_3_ measurements (65). This will provide more data for further analysis that connects air quality and climate. However, these policies often overlook the indirect climate implications of NH_3_-driven aerosol formation in the UTLS. While reducing agricultural NH_3_ emissions can improve air quality, our findings indicate that it also leads to a depletion of the UTLS aerosol layer, which can have significant implications for the Earth’s radiation balance. Incorporating our findings into IPCC (The Intergovernmental Panel on Climate Change) socioeconomic models can help refine projections of aerosol–cloud–climate interactions and guide policies that balance agricultural productivity with climate goals, contributing to the objectives of the UNFCCC Paris Agreement and promoting environmental and societal sustainability (66, 67).

## Methods

### EMAC Model.

The EMAC (ECHAM/MESSy Atmospheric Chemistry) model is a numerical framework designed to simulate global atmospheric chemistry and climate interactions. It integrates various submodels that represent atmospheric processes and their interactions with land, oceans, and anthropogenic influences (68). The dynamical core is the ECHAM5 atmospheric circulation model, which is linked with the second version of the Modular Earth Submodel System (MESSy2) to facilitate the integration of multi-institutional computer codes (49). To ensure realistic atmospheric transport conditions, meteorological prognostic variables are nudged toward ECMWF ERA-5 reanalysis data through Newtonian relaxation. Atmospheric chemical kinetics are computed online at each model time step using the MIM chemistry mechanism (69). EMAC follows the 1957 World Meteorological Organization definition of the tropopause, which is the lowest altitude at which the temperature lapse rate decreases to $2\;\text{K}\;{\text{km}}^{-1}$ or less, provided that the average lapse rate within the next 2 km does not exceed this threshold (70).

For this study, we use EMAC (ECHAM5 version 5.3.02, MESSy version 2.55.2) at a T63L90 resolution to simulate the period from January 2003 to December 2019. The simulation period allows for direct comparison with the satellite retrievals by the MIPAS and IASI instruments. The model employs 90 vertical hybrid levels extending from the surface to approximately 80 km altitude (0.01 hPa) and applies a spherical truncation of T63, corresponding to a horizontal grid resolution of 1.875^°^× 1.875^°^ at the equator. A 10-min time step is used, with model output at hourly intervals. All conditions are identical in both simulations, with the only difference being that anthropogenic NH_3_ emissions are turned off in one of them.

The simulation code encompasses multiple submodels: i) GMXe that handles aerosol microphysics (71), ii) NAN that is responsible for nucleation mechanisms (72), iii) IONS that simulates ion pair production due to cosmic rays and radon decay (72), iv) AEROPT that calculates aerosol optical properties (73), v) MECCA, dedicated to gas-phase chemistry (74), vi) JVAL for photochemistry rates (75), and vii) DRYDEP, which simulates dry deposition using the big-leaf approach (76, 77). Dry deposition of NH_3_ introduces additional uncertainty due to the complexities of bidirectional exchange. Sensitivity simulations were carried out to account for the uncertainties in NH_3_ budget (*SI Appendix*, Figs. S1–S3).

In this study, the CONVECT submodel is employed to parameterize convection using the Tiedtke scheme (78) with Nordeng closure (79), a standard configuration for T63 resolution (80). The LNOX submodel calculates real-time ${\text{NO}}_{\text{x}}$ emissions from lightning activity (81), applying the parameterization developed by Grewe et al. (82), which correlates flash frequency with updraft velocity.

Our model computes the molality of semivolatile compounds and the equilibrium states of binary solutions, considering both stable and metastable phases (83). ${\text{HNO}}_{3}$ is produced by the oxidation of lightning ${\text{NO}}_{\text{x}}$. In the UT, transport processes primarily influence production rather than local reactions (52). ${\text{H}}_{2}{\text{SO}}_{4}$ is mainly produced through the oxidation of sulfur dioxide (49).

**Fig. 5.**
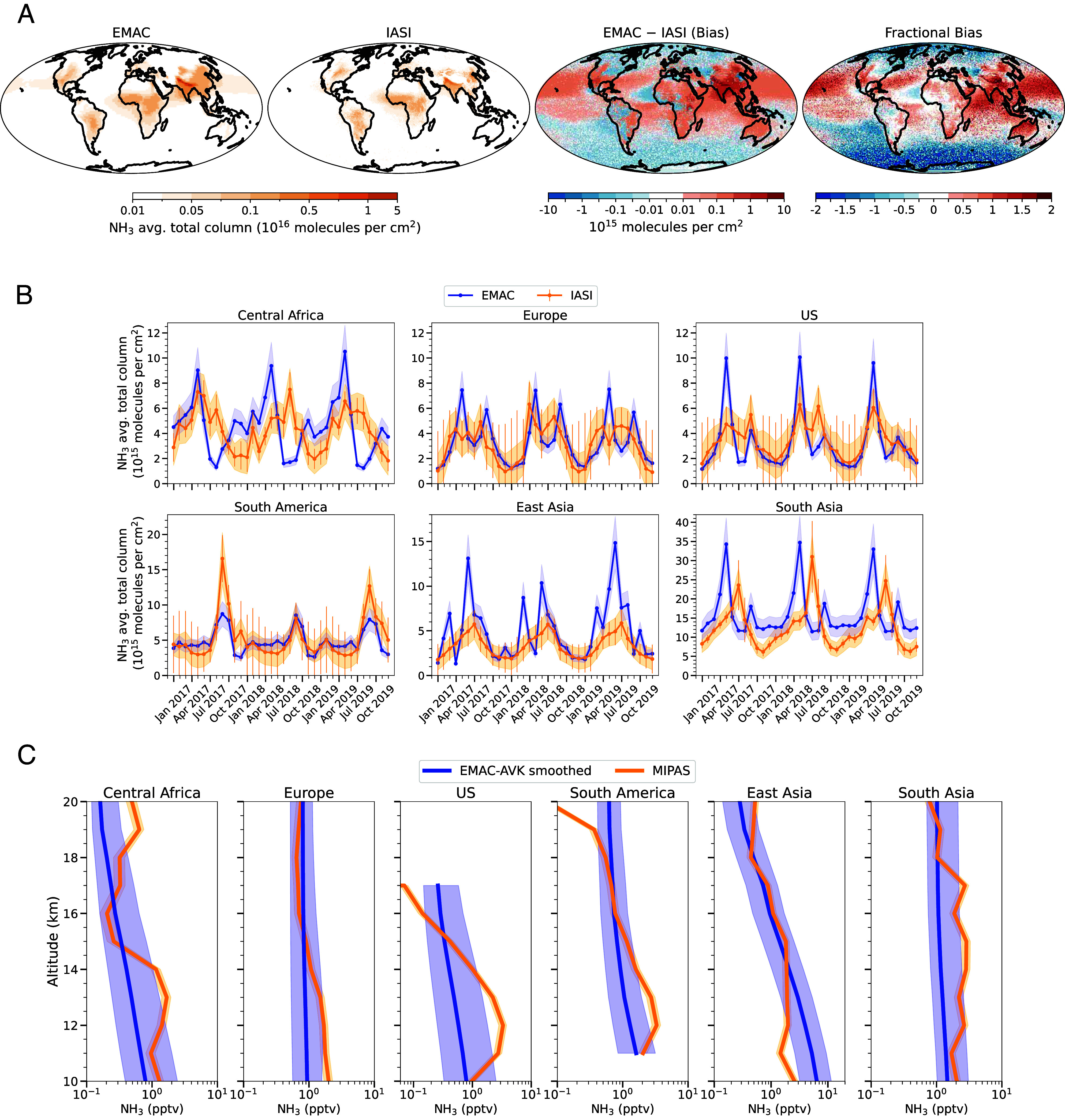
(*A*) Spatial distribution of NH_3_ total column concentrations (0 to 20 km) from EMAC simulations compared to IASI satellite retrievals, averaged over January 2017–December 2019. (*B*) Temporal validation of monthly NH_3_ column averages at key emission hotspot regions, showing consistency between EMAC and IASI observations from January 2017–December 2019. The light blue shaded areas represent the temporal SD in EMAC. The orange vertical lines and shaded areas represent random and systematic uncertainties in IASI observations, respectively. (*C*) Vertical NH_3_ profiles from EMAC simulations, smoothed using averaging kernels, compared against MIPAS retrievals. The blue lines and shaded areas represent the medians and the interquartile ranges for 2003–2011 in EMAC, respectively. The orange lines and shaded areas represent the median and the uncertainty in MIPAS measurements, respectively. According to ref. 40, the uncertainty in MIPAS data is estimated as ±5 pptv (single profile precision) and ±15% (accuracy).

In Fig. 5, NH_3_ distributions across space, time, and altitude generated by the EMAC model are compared against satellite measurements, with a focus on the UTLS. The MIPAS (Michelson Interferometer for Passive Atmospheric Sounding) instrument aboard the ENVISAT satellite retrieves vertical profiles of NH_3_ by detecting its infrared emissions. MIPAS is a high-resolution mid-infrared emission limb sounder designed to measure NH_3_ volume mixing ratios, along with a broad range of other atmospheric trace species (84). We construct synthetic averaging kernels to account for vertical resolution effects when averaging model data for comparison with MIPAS retrieved values. We use the vertical resolution, defined as the altitude grid spacing over the averaging kernel diagonal, to generate Gaussian-shaped weighting functions at different altitudes. Each Gaussian function is centered at a given altitude, with its half-width determined by the corresponding vertical resolution value. The model profile is then convolved with these Gaussian functions, effectively smoothing it to match the vertical sensitivity of the retrieval. We also compare total column concentrations against retrievals by the IASI (Infrared Atmospheric Sounding Interferometer) instrument aboard the MetOp satellites that measures NH_3_ total column concentrations globally using infrared spectroscopy (85–88).

Fig. 5*A* compares the spatial distribution of global NH_3_ total column concentrations from EMAC simulations and IASI observations, averaged over 2017–2019. The comparison identifies major hotspots in South and East Asia, Central Africa, Europe, South America, and the United States. Fig. 5*B* illustrates monthly averages of total column NH_3_ from EMAC and IASI. While the model overestimates NH_3_ concentrations over South and East Asia, the normalized mean biases are within ±10% in all other hotspot regions. Both globally and across all hotspot regions, Pearson correlation coefficients range from 0.6 to 0.85, and 60 to 91% of simulated data fall within a factor of two of the observations (*SI Appendix*, Table S1). Fig. 5*C* compares the vertical profiles of EMAC kernel-adjusted NH_3_ median profiles and the equivalent MIPAS retrievals for 2003–2011 in the UTLS over hotspot regions. Simulated values broadly encompass the observations and their associated uncertainties.

The model has also been evaluated against AIRS (Atmospheric Infrared Sounder) NH_3_ global measurements (89–91) from the Aqua satellite within the 4 to 5.5 km altitude range. EMAC reproduces the observed concentrations with normalized mean biases within ±8%, Pearson correlation coefficients above 0.7 across all regions, and more than 70% of simulated data falling within a factor of two of the observations (*SI Appendix*, Table S1 and Fig. S5).

Simulated ${\text{NH}}_{4}^{+}$ concentrations were also compared with aircraft observations from the NASA ATom (Atmospheric Tomography) mission (92), covering continental North America, the Pacific, Atlantic, and Southern Ocean (*SI Appendix*, Fig. S6). The model captures the observed vertical distributions of ${\text{NH}}_{4}^{+}$ across ATom campaigns, with simulated concentrations within the observed interquartile range (*SI Appendix*, Fig. S7).

Finally, the simulated AOD and CCN values were compared to MODIS (MODerate Resolution Imaging Spectroradiometer) (93) and E3SM (Energy Exascale Earth System Model) (10), respectively, with consistent regional patterns between models (*SI Appendix*, Figs. S8 and S9). The model has also been evaluated extensively over the Asian monsoon region (27).

### CEDS Inventory.

Trace gas emissions, including NH_3_, are adopted from the CEDS inventory (62). CEDS includes default global emission estimates for each compound, based on a bottom–up approach. These estimates are scaled to match global inventories by sector and fuel type and subsequently refined using regional or national inventories that incorporate more accurate local data. CEDS involves normalized spatial distribution proxies to scale yearly country-level emission estimates onto a global $0.5^{{}^{\circ}}$×$0.5^{{}^{\circ}}$ grid. These gridded emission fluxes are aggregated into sectors and distributed over a 12-mo period. The spatial distribution and magnitude of the simulated NH_3_ emissions are shown in *SI Appendix*, Fig. S10, and the sectoral contributions are illustrated in *SI Appendix*, Fig. S11.

Compared to other inventories, such as EDGAR and ECLIPSE, CEDS adopts a mosaic approach that integrates activity and emission input data from multiple sources-including EDGAR, ECLIPSE, and detailed regional and national inventories, such as SMoG-India. This approach reduces uncertainty and produces global emission estimates that are historically consistent and aligned with contemporary country-level data from 1970 to 2017, particularly in regions undergoing rapid energy and policy shifts (62, 94, 95).

### NPF and Aerosol Submodels.

The EMAC model incorporates the NAN (New Aerosol Nucleation) submodel (72) to represent NPF. NAN estimates nucleation rates using parameterizations derived from the CERN CLOUD experiment, specifically, neutral and ion-induced binary nucleation involving H_2_SO_4_ and H_2_O (47) and ternary nucleation of NH_3_–H_2_SO_4_–H_2_O (47), and a synergistic nucleation mechanism involving NH_3_–H_2_SO_4_–HNO_3_–H_2_O (35). The integration of these NPF parameterizations within EMAC is described in detail by Ehrhart et al. (72). Details on the implementation of the most recently published CLOUD parameterization for synergistic nucleation (35) are provided by Xenofontos et al. (27).

The IONS submodel calculates atmospheric ion pair production rates and steady-state ion concentrations, considering galactic cosmic rays and radon decay. It provides real-time ion pair production rates for ion-induced nucleation while accounting for losses through ion–ion recombination and aerosol scavenging. The NAN and IONS submodels have been evaluated by Ehrhart et al. (72).

The GMXe (Global Modal-aerosol eXtension) submodel (71) simulates aerosol dynamics through a full thermodynamic treatment of gas/aerosol partitioning using the ISORROPIA-II model (83). GMXe represents the aerosol size distribution as seven log-normal modes—four hydrophilic and three hydrophobic. Aerosol number concentration and mass for each component are prognostically calculated, assuming a constant geometric SD for each mode. The aerosol size distribution follows:

$$n\left(\ln r\right)=\sum_{i=1}^{7}\frac{N_{i}}{\sqrt{2\pi }\ln\sigma_{i}}\exp\left(-\frac{{\left(\ln r-\ln{\tilde{r}}_{i}\right)}^{2}}{2\ln^{2}\sigma_{i}}\right),$$

where each mode (i) is characterized by its number concentration $\left(N_{i}\right)$, number median radius $\left({\tilde{r}}_{i}\right)$, and geometric SD $\left(\sigma_{i}\right)$. The four hydrophilic modes span the full aerosol size range: i) nucleation (<10 nm), ii) Aitken (10–100 nm), iii) accumulation (100 to 1,000 nm), and iv) coarse (>1,000 nm). The three hydrophobic modes mirror these size ranges, encompassing Aitken, accumulation, and coarse modes (71). In our simulations, σ=1.59 for the nucleation mode and both (hydrophilic and hydrophobic) Aitken modes, σ=1.49 for both accumulation modes, and σ=1.7 for both coarse modes. Composition is uniform (internal mixing) within modes, but different modes exhibit compositional variation (external mixing).

Coagulation in GMXe follows the method in ref. 96, with coagulation coefficients derived from Brownian motion theory based on the original work of Fuchs (97). The coagulation matrix accommodates varying species numbers within each mode. Coagulation processes transfer aerosol particles from smaller to larger modes and hydrophobic to hydrophilic modes (71).

## Supplementary Material

Appendix 01 (PDF)

## Data Availability

The EMAC (ECHAM/MESSy Atmospheric Chemistry) model is continuously developed and used by a consortium of institutions. Members of institutions within the MESSy consortium are granted a license to use MESSy and access its source code. Institutions can join the consortium by signing the MESSy Memorandum of Understanding. Further information is available on the MESSy consortium website (https://www.messy-interface.org) (98). The results presented in this paper were produced using MESSy version 2.55.2. A permanent identifier (DOI: 10.5281/zenodo.14875637) (99) has been assigned in Zenodo under the “CERN CLOUD experiment community.” This includes the EMAC configuration files, namelist setup, chemical mechanisms, and details on the emissions setup. Additionally, the complete dataset used in the figures will be made available upon finalization for publication to ensure long-term accessibility and facilitate reproducibility.
