# Supplementary material for: Global impact of anthropogenic NH3 emissions on upper tropospheric aerosol formation
Source: Proc Natl Acad Sci U S A. 2025 Oct 27;122(44):e2506658122. doi: 10.1073/pnas.2506658122 (PMC12595474; doi:10.1073/pnas.2506658122)
Supplement: Supplementary file 1 — Appendix 01 (PDF) [file pnas.2506658122.sapp.pdf]

Supporting Information (SI) for:
Global Impact of Anthropogenic NH_3 Emissions on Upper Tropospheric Aerosol Formation

Xenofontos et al.

This PDF file includes: Figures S1–S11, and Table S1.

Model Uncertainty: $J_{1.7}$ and AOD vertical profiles

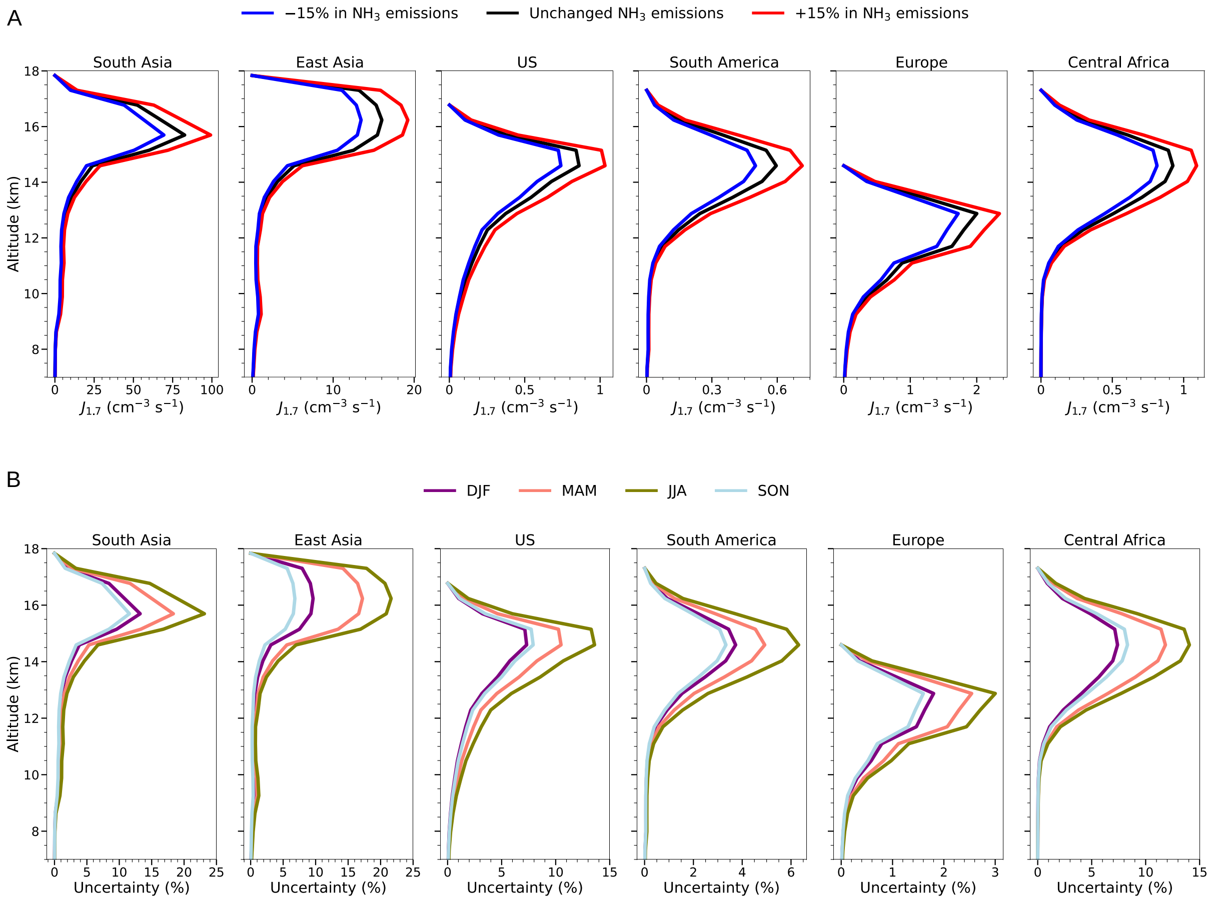

Fig. S1. *(A)* Vertical profiles of the total nucleation rate at 1.7 nm diameter ($J_{1.7}$) in the upper troposphere-lower stratosphere over NH_3 emission hotspots. The uncertainties are defined as the relative differences between emission-perturbed and unperturbed EMAC simulations. The black line shows the profile for the unperturbed scenario with unchanged NH_3 emissions, while the red and blue lines represent the scenarios with +15% and –15% changes in global NH_3 emissions, respectively. *(B)* Vertical profiles of the uncertainty in $J_{1.7}$ in the upper troposphere–lower stratosphere over NH_3 emission hotspots. The uncertainties are derived from region- and season-specific perturbations in NH_3 emissions. The emission perturbation magnitudes were determined by scaling NH_3 emissions according to the EMAC-to-IASI ratio in each hotspot region for each month. Uncertainties are expressed as percentage changes relative to the unperturbed scenario. Results are shown for DJF (purple), MAM (red), JJA (green), and SON (blue).

Fig. S1 illustrates the sensitivity in the simulated upper tropospheric nucleation rate at 1.7 nm diameter ($J_{1.7}$) based on (A) global and (B) region- and season-specific perturbations in NH_3 emissions. These perturbations are propagated through the model simulations, including all associated feedbacks. Panel A shows that increasing global NH_3 emissions by 15% (red lines) enhances the total UTLS $J_{1.7}$ in all regions, with particularly strong responses in South and East Asia. Reducing global NH_3 emissions by 15% (blue lines) suppresses $J_{1.7}$ in all high-emission regions. The variability is less pronounced in lower altitudes. Panel B illustrates the resulting uncertainty in $J_{1.7}$ due to region- and season-specific NH_3 emission perturbations. Uncertainty peaks during the summer season (JJA) across all hotspot regions, with the largest values in South and East Asia reaching up to 25%. Elevated uncertainties are also observed during spring, while winter and autumn show minimal sensitivity across all hotspot regions. This

highlights the importance of accurate regional NH_3 emission inventories and improved observational constraints during convective periods.

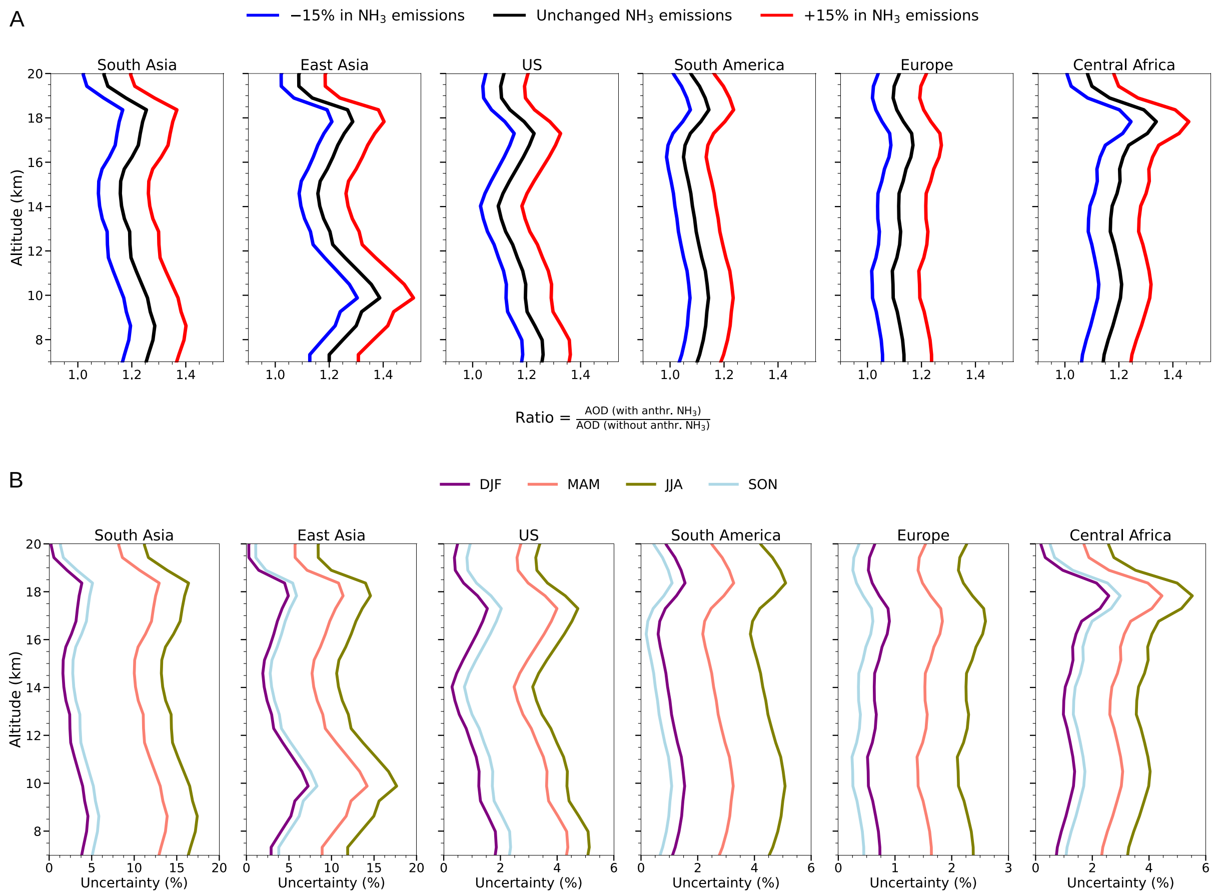

Fig. S2. *(A)* Vertical profiles of the aerosol optical depth (AOD) ratio, defined as AOD (at 550 nm) with anthropogenic NH_3 relative to AOD without. The uncertainties are defined as the relative differences between emission-perturbed and unperturbed EMAC simulations. The black line corresponds to the unperturbed scenario, and the red and blue lines show the effect of +15% and –15% changes in global NH_3 emissions, respectively. *(B)* Vertical profiles of the uncertainty in AOD ratio over NH_3 emission hotspots. The uncertainties are derived from region- and season-specific perturbations in NH_3 emissions. The emission perturbation magnitudes were determined by scaling NH_3 emissions according to the EMAC-to-IASI ratio in each hotspot region for each month. Uncertainties are expressed as percentage changes relative to the unperturbed scenario. Results are shown for DJF (purple), MAM (red), JJA (green), and SON (blue).

Fig. S2 illustrates the sensitivity in the simulated upper tropospheric aerosol optical depth (AOD) ratio, defined as AOD (at 550 nm) with anthropogenic NH_3 emissions relative to AOD without, based on (A) global and (B) region- and season-specific perturbations in NH_3 emissions. These perturbations are propagated through the model simulations, including all associated feedbacks. Panel A shows that these changes in global NH_3 emissions modulate AOD in the upper troposphere, with elevated NH_3 leading to higher AOD (red lines) and reduced NH_3 lowering AOD ratios (blue lines) over all emission hotspots. Panel B illustrates the resulting uncertainty in UT AOD ratio due to region- and season-specific NH_3 emission perturbations. This uncertainty peaks during the summer season (JJA) across all hotspot regions. Elevated uncertainties are also observed during spring, while winter and autumn show minimal sensitivity across all hotspot regions.

Model Uncertainty: CCN and AOD

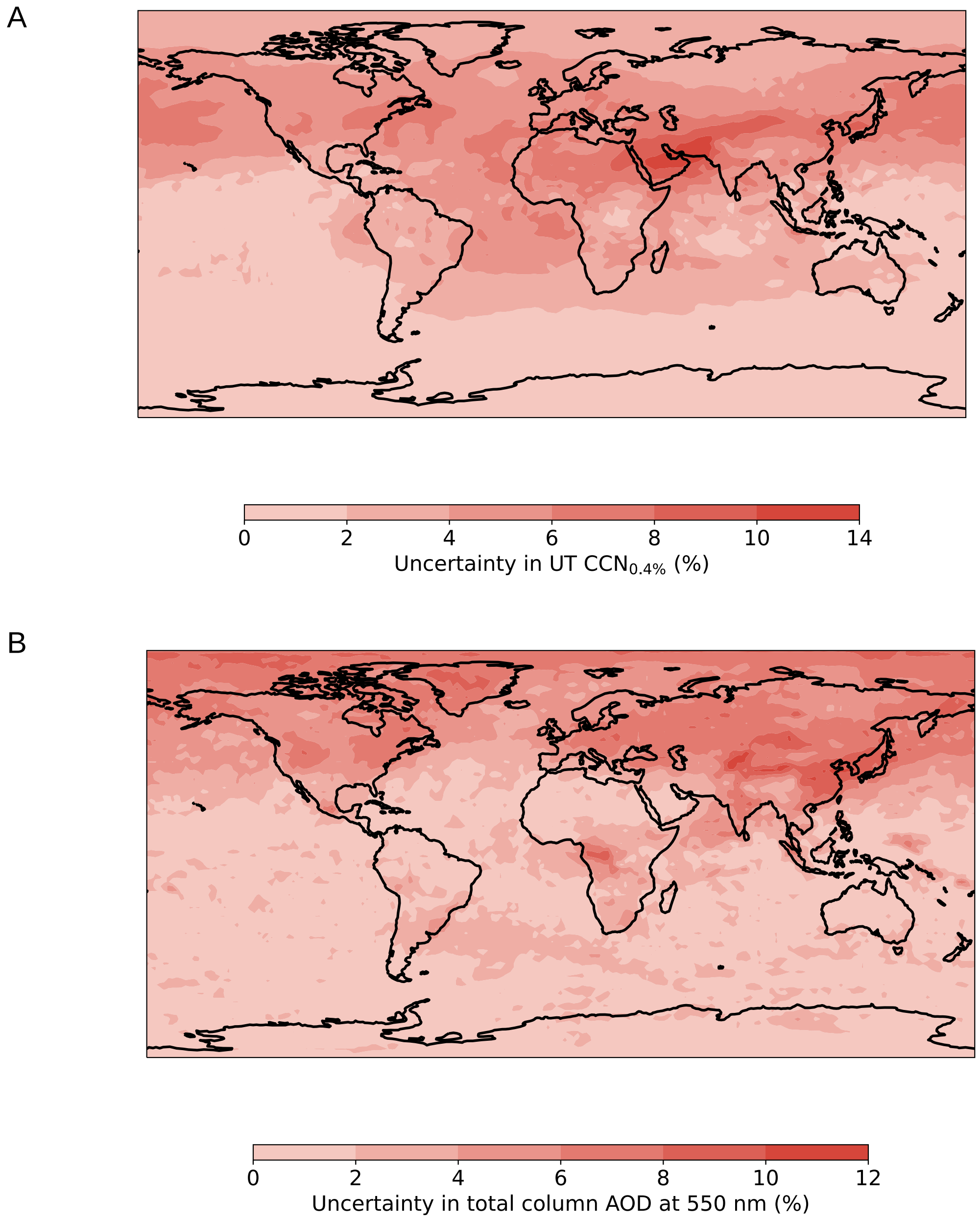

Fig. S3. *(A)* Global maps of upper tropospheric cloud condensation nuclei at 0.4% supersaturation (UT $CCN_{0.4\%}$) and *(B)* total column AOD at 550 nm, where the percentage change relative to the unperturbed scenario represents the uncertainty due to $\pm 15\%$ global NH_3 emission perturbations.

To assess the overall model uncertainty, taking into account the complex interplay of factors of variability, we conducted a sensitivity test by perturbing global NH_3 emissions by $\pm 15\%$ (based on the statistical analysis of the global model normalized mean bias in Table S1) and propagating through the model and submodel components the resulting changes. The overall uncertainty in upper tropospheric cloud condensation nuclei at 0.4% supersaturation (UT $CCN_{0.4\%}$) and total column aerosol optical depth (AOD) at 550 nm is shown in Fig. S3. The relative uncertainty remains within 15% globally, with the largest sensitivity in regions influenced by intense anthropogenic NH_3 sources and strong dynamical variability, such as South and East Asia. In these regions, the uncertainties in $CCN_{0.4\%}$ and AOD can reach up to 30% at certain altitudes during the summer monsoon season.

Temporal Trends in Emissions and Nucleation Rate ($J_{1.7}$)

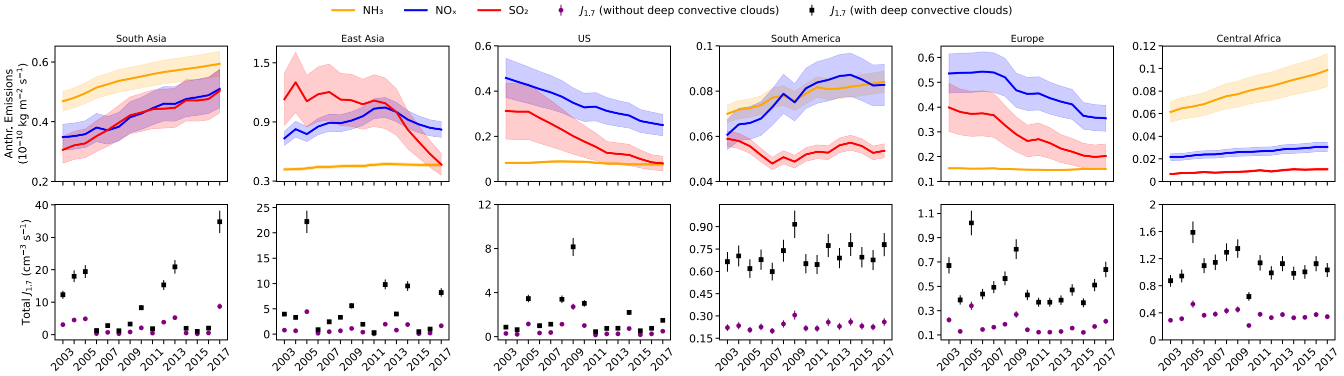

Fig. S4. Simulated temporal trends (2003–2017) of spatially averaged anthropogenic emissions of NH_3 (orange), NO_x (blue), and SO_2 (red), from the Community Emissions Data System (CEDS) for all sectors, and corresponding nucleation rates at 1.7 nm diameter ($J_{1.7}$) in the upper troposphere–lower stratosphere over NH_3 emission hotspots. The top panels show annual mean anthropogenic emissions, with solid lines indicating regional means and shaded areas representing temporal standard deviations. The bottom panels display total $J_{1.7}$ values (annual mean) simulated with (black squares) and without (purple circles) the presence of deep convective clouds. Error bars reflect the temporal standard deviation.

Fig. S4 shows annual mean trends (2003–2017) in anthropogenic NH_3, NO_x, and SO_2 emissions from the Community Emissions Data System (CEDS), with concomitant $J_{1.7}$ (nucleation rate at 1.7 nm diameter) from EMAC in the upper troposphere–lower stratosphere (UTLS). Individual species emissions in certain regions exhibit temporal trends, with increasing NH_3 fluxes in South Asia, and declining SO_2 and NO_x in Europe and the US due to emission controls. The response to these trends of $J_{1.7}$ is influenced by a complex interplay of the subsequent reactions producing multiple nucleation precursors (e.g. oxidation of SO_2 to H_2SO_4), the presence of natural emissions (such as lightning NO_x), and transport dynamics, in particular convective updrafts. The variability in $J_{1.7}$ is thus not solely modulated by the trend of individual species emissions over each region, and without consistent long-term trend across all regions. The presence of deep convective clouds (bottom row) substantially enhances $J_{1.7}$ values across all regions; thus it is expected that, other than emission trends, meteorological factors such as monsoon duration and intensity, play an important role in year-to-year temporal variability.

Statistical Evaluation of Simulated NH_3

A

IASI

	Global	South Asia	East Asia	US	South America	Europe	Central Africa
**NMB**	14%	27%	50%	-5.7%	-6.9%	6.4%	6.2%
**Pearson *R***	0.8	0.85	0.6	0.6	0.8	0.7	0.6
**PF2**	81%	70%	60%	70%	87%	85%	91%

B

AIRS

	Global	South Asia	East Asia	US	South America	Europe	Central Africa
**NMB**	-8%	8%	8%	4%	2%	-8%	7%
**Pearson *R***	0.79	0.75	0.76	0.81	0.73	0.96	0.72
**PF2**	75%	76%	70%	70%	77%	76%	70%

Table S1. Statistical evaluation of EMAC-simulated NH_3 concentrations against satellite observations from *A)* IASI and *B)* AIRS over the emission hotspot regions. These regions are indicated in Fig. 1*B*. The evaluation metrics (first column) are the Normalized Mean Bias (NMB), Pearson correlation coefficient (*R*), and the percentage of simulated data within a factor of two (PF2) of the observations.

To quantify model–observation agreement, we computed the normalized mean bias (NMB) between EMAC-simulated and IASI-retrieved total column NH_3 concentrations. We also computed the Pearson correlation coefficient (R) to assess linear agreement between model and observations, and the percentage of simulated values falling within a factor of two of the observations. Table S1 lists statistical comparison metrics evaluating EMAC-simulated NH_3 concentrations against satellite retrievals from A) IASI and B) AIRS, both globally and across NH_3 emission hotspots. For IASI, the global NMB is $+14\%$, with regional NMBs within $\pm 10\%$ except South and East Asia, where EMAC exhibits a substantial positive bias of $+27\%$ and $+50\%$, respectively. Pearson correlation coefficients (R) range from 0.6 to 0.85 across regions, with a global R of 0.8. The percentage of simulated values falling within a factor of two of the observations (PF2) ranges from 60% (East Asia) to 91% (Central Africa). Regarding AIRS, the global NMB is marginally negative (–8%), suggesting a modest underestimation by EMAC. Regional biases are all within $\pm 8\%$. Pearson correlation coefficients are equivalently high, with Europe showing the strongest agreement ($R = 0.96$) and other regions ranging from 0.72 to 0.81. The PF2 values for AIRS are consistently above 70% across all regions (75% globally).

Additional Model Evaluation: AIRS

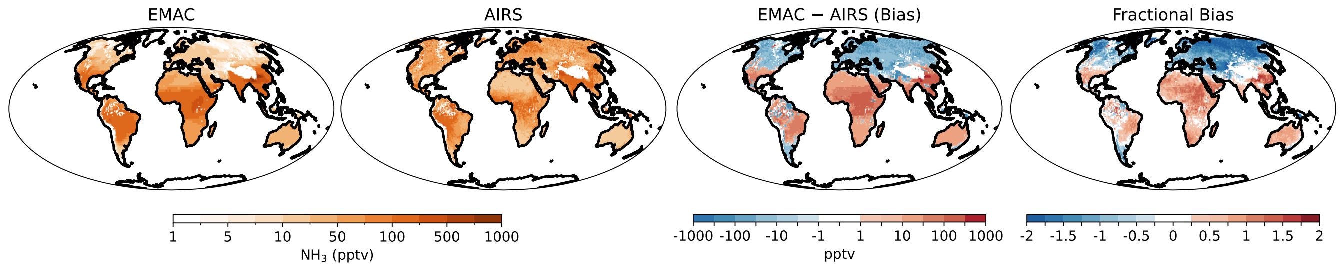

Fig. S5. Spatial maps of NH_3 amount in EMAC simulations and AIRS satellite data, averaged over January 2007 to December 2011 within the 4–5.5 km altitude range, including absolute (EMAC−AIRS), and fractional bias.

Additional Model Evaluation: ATom

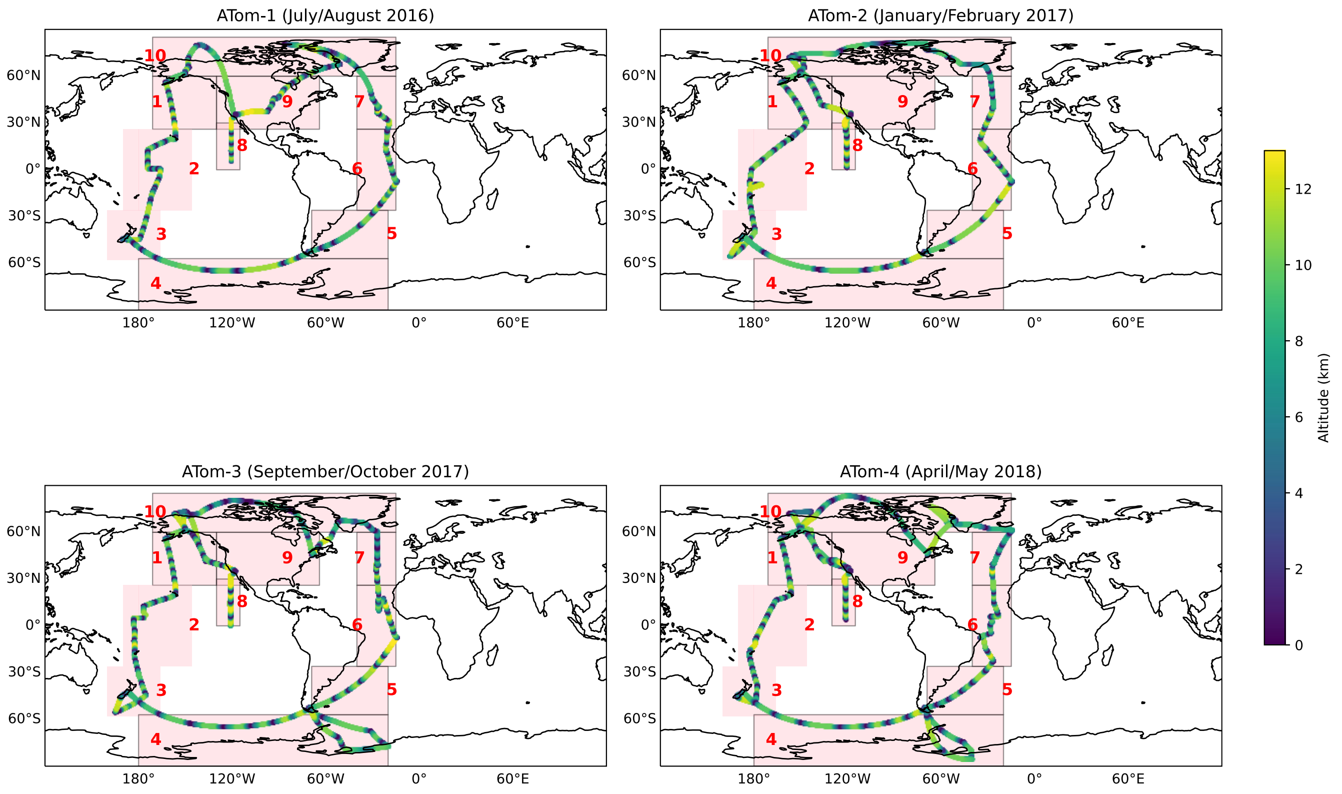

Fig. S6. Flight tracks from the four Atmospheric Tomography (ATom) campaigns. Flight path color represents sampling altitude. For analysis (Fig. S7), the flight paths are grouped into ten separate sectors: North Pacific (1), Pacific Tropics (2), South Pacific (3), Southern Ocean (4), South Atlantic (5), Atlantic Tropics (6), North Atlantic (7), East Pacific (8), Continental US/CA (9), and Global North (10).

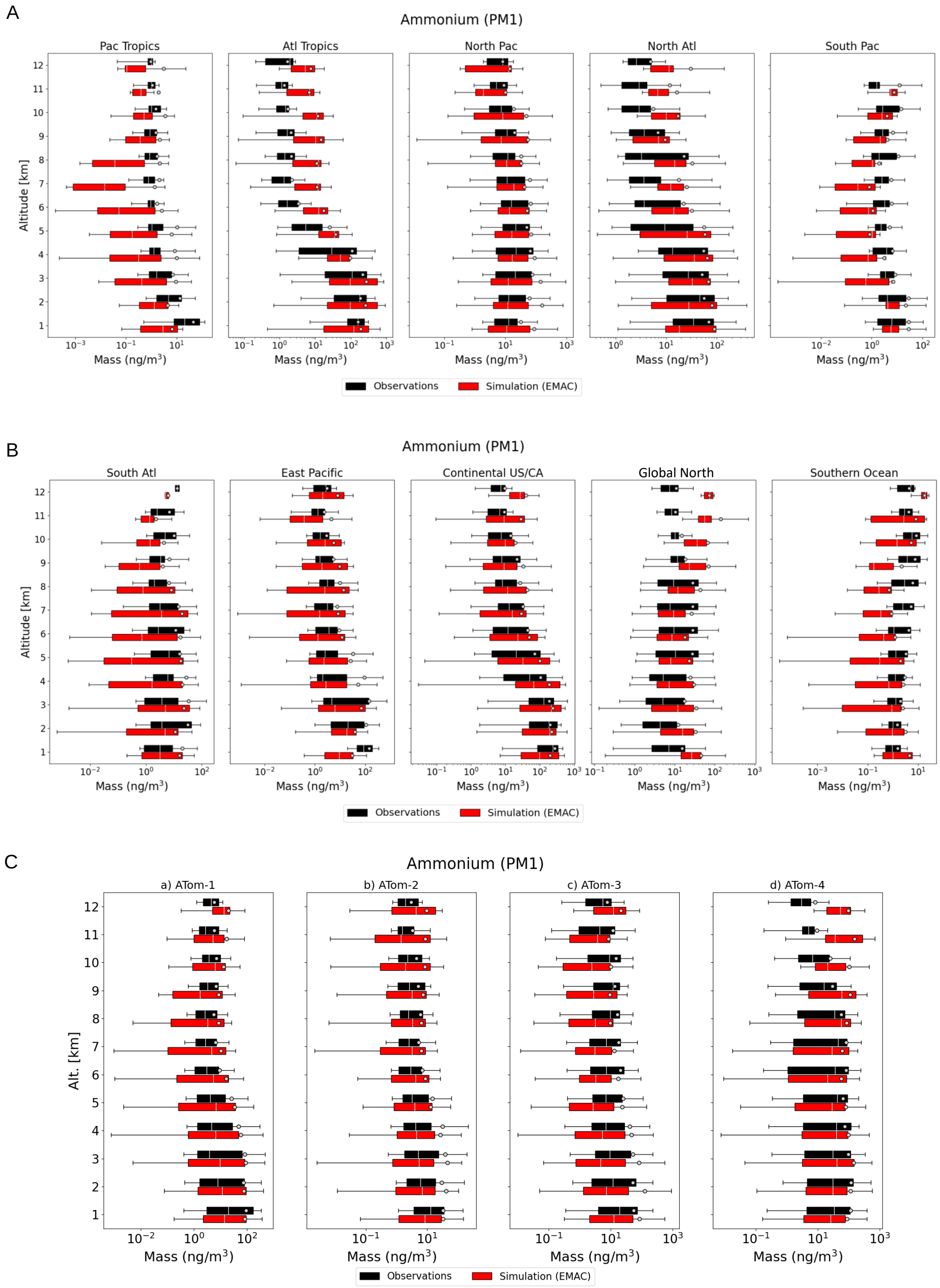

Fig. S7. Comparison of simulated (red) and observed (black) mass concentration of ammonium aerosol (PM_1) from the ATom campaign over continental North America, the Pacific, Atlantic, and Southern Ocean (flight tracks are shown in Fig. S6). Model data are compared to all flights during ATom between July 2016 and May 2018. During the simulation, model data are sampled at the grid boxes corresponding to the actual flight tracks, incorporating the specific dates and times of each flight to ensure close correspondence with observed measurements. Grey points indicate mean values, white vertical lines represent medians, boxes show the interquartile range, and whiskers denote the 5th and 95th percentiles. Panels *(A)* and *(B)* show the comparison by region, averaged across all four ATom campaigns, and panel *(C)* shows the comparison by individual campaigns (ATom-1 to ATom-4), averaged across all regions.

Additional Model Evaluation: MODIS

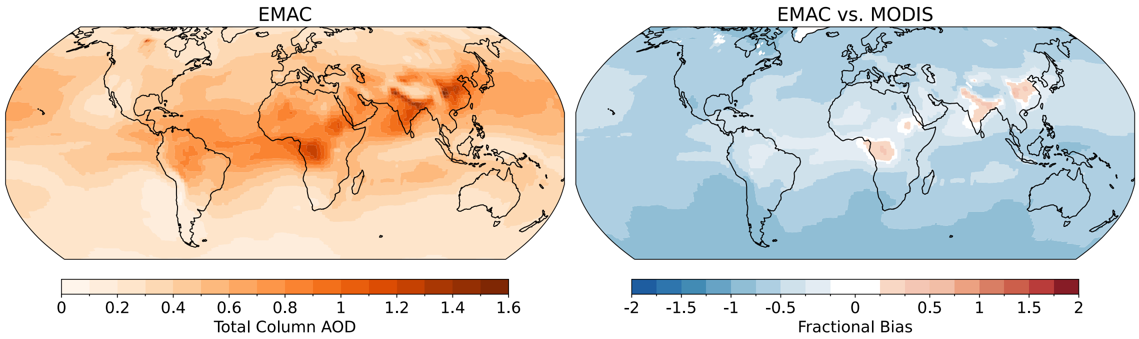

Fig. S8. Total column aerosol optical depth (AOD) at 550 nm and model bias averaged over January 2016 to December 2017. Simulated total column AOD from the EMAC model (left). Fractional bias between EMAC-simulated AOD and MODIS satellite observations (right). Positive values (in red) indicate model overestimation, and negative values (in blue) indicate underestimation.

We assess the simulated total column aerosol optical depth (AOD) at 550 nm by comparing it with satellite observations from the MODerate Resolution Imaging Spectroradiometer (MODIS) aboard the Terra platform. For this comparison, we use the MODIS Level 3 product (1), which employs the Deep Blue retrieval algorithm (2). Deviations fall within ±50% in South and East Asia, Central Africa, South America, and parts of Europe (Fig. S8).

Additional Model Evaluation: EMAC vs. E3SM

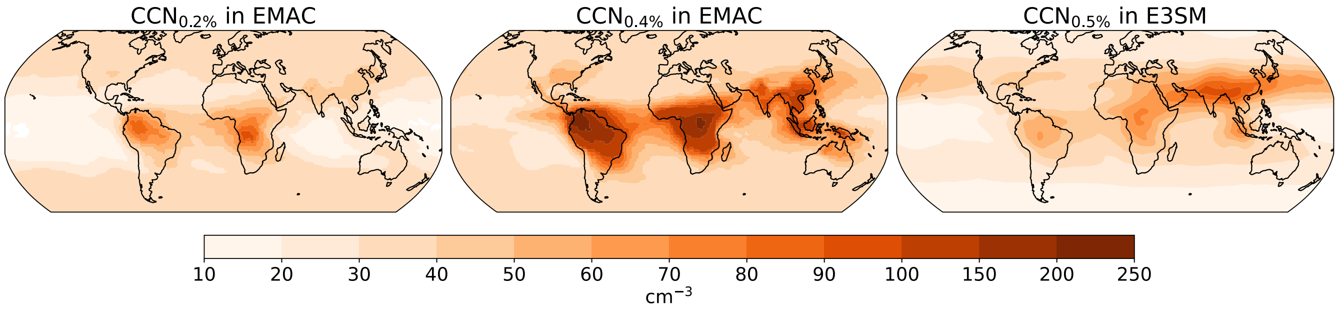

Fig. S9. Annual mean cloud condensation nuclei (CCN) concentrations at 13 km altitude in 2016 from EMAC and E3SM simulations at different supersaturations. Left and center panels show CCN concentrations at 0.2% and 0.4% supersaturation ($CCN_{0.2\%}$ and $CCN_{0.4\%}$) from EMAC simulations, respectively. The right panel shows CCN concentrations at 0.5% supersaturation ($CCN_{0.5\%}$) from E3SM simulations, which include the NH_3–H_2SO_4–HNO_3–H_2O nucleation parameterization following Zhao et al. (3). Color shading indicates CCN number concentrations in cm^{-3}.

Fig. S9 compares simulated cloud condensation nuclei (CCN) concentrations at supersaturations of 0.2% ($CCN_{0.2\%}$) and 0.4% ($CCN_{0.4\%}$) from EMAC with CCN concentrations at 0.5% supersaturation ($CCN_{0.5\%}$) from the E3SM (Energy Exascale Earth System Model) model (3) at 13 km altitude. Although the absolute values differ as expected due to variations in supersaturation thresholds and model formulations, the spatial patterns exhibit agreement. EMAC predicts higher $CCN_{0.4\%}$ concentrations over Central Africa and South America relative to E3SM, while AOD remains lower than MODIS observations. This may indicate better overall performance of EMAC in these regions. However, further comparisons between models are needed to understand the sources of these differences. Across the US and Europe, the two models agree within 50%. Both models consistently show the highest CCN concentrations over Central Africa, South and East Asia, and South America.

NH_3 surface emissions

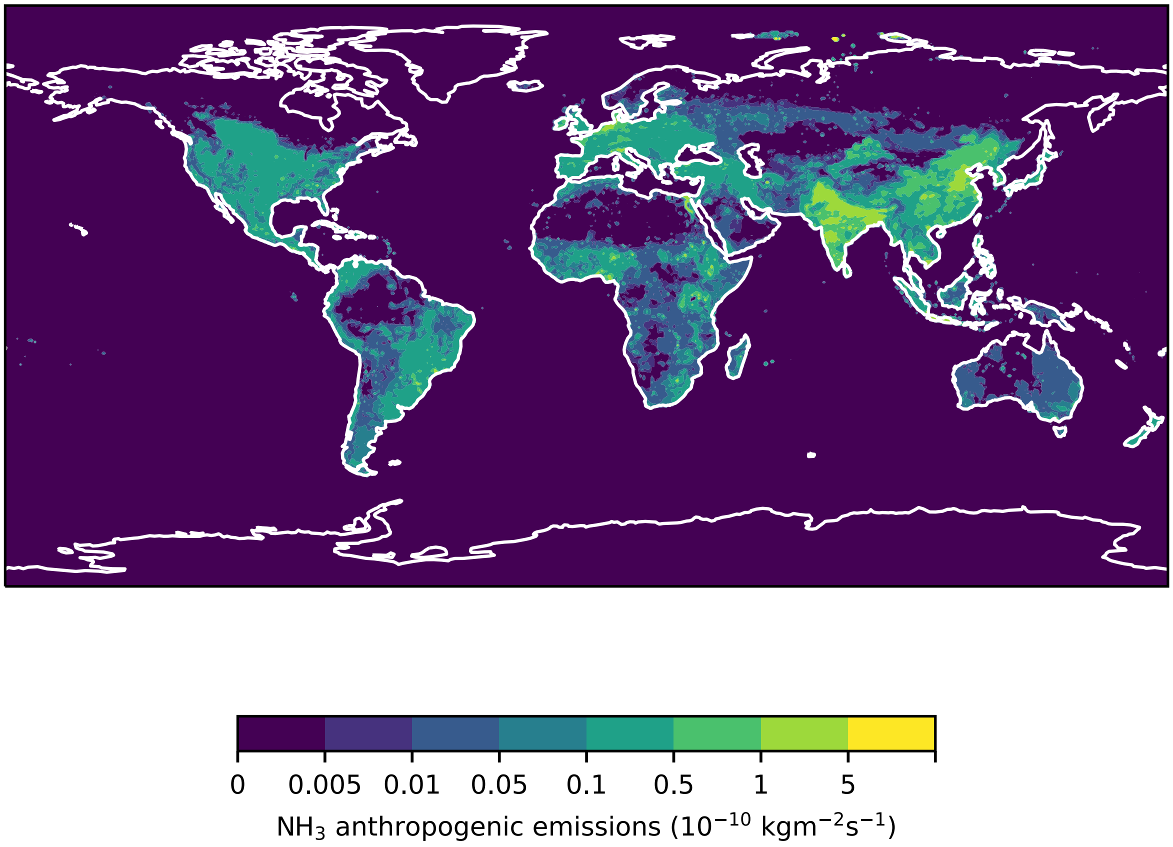

Fig. S10. Global map of surface anthropogenic emissions of NH_3, averaged from January 2003 to December 2017 for all sectors based on the Community Emissions Data System (CEDS), integrated into the EMAC model.

NH_3 Emissions by Sector

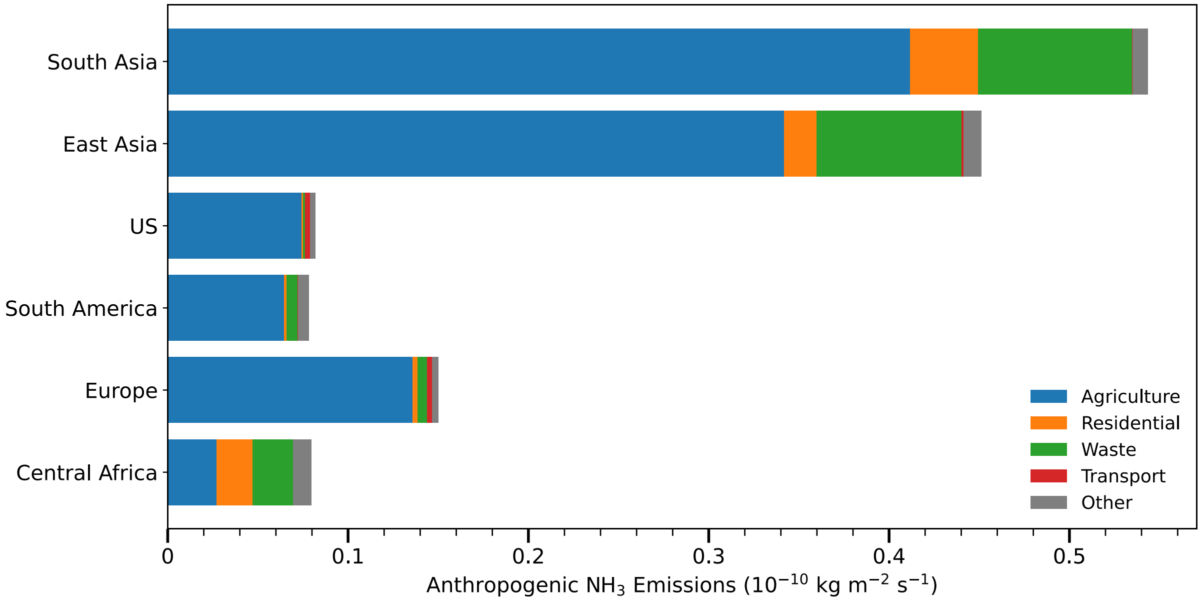

Fig. S11. Anthropogenic NH_3 emissions by sector and region, integrated into the EMAC model, based on the Community Emissions Data System (CEDS). Emissions are categorized into agriculture (in blue), residential (in orange), waste (in green), transport (in red), and other sectors (in gray), averaged over the years 2003–2017.

Fig. S11 presents the CEDS anthropogenic NH_3 emissions by sector (agriculture, residential, waste, transport, other) for different regions as multi-year averages. Anthropogenic NH_3 emissions are primarily driven by agricultural sources in all regions, with South Asia and East Asia collectively accounting for the majority of global emissions. In South and East Asia, agriculture alone accounts for more than 70% of the total regional NH_3 emissions, mostly complemented by the waste and residential sectors. In contrast, emissions in Central Africa show a more uniform sectoral contribution, with agriculture, residential, and waste sectors each contributing almost equally to the total regional emissions. While transport and other sectors constitute a relatively small fraction of total emissions in the CEDS inventory, their role may be more pronounced in urban areas.

1. S Platnick, et al., MODIS cloud optical properties: User guide for the Collection 6 Level-2 MOD06/MYD06 product and associated Level-3 Datasets. *Version* **1**, 145 (2015).
2. NC Hsu, SC Tsay, MD King, JR Herman, Aerosol properties over bright-reflecting source regions. *IEEE transactions on geoscience remote sensing* **42**, 557–569 (2004).
3. B Zhao, et al., Global variability in atmospheric new particle formation mechanisms. *Nature* **631**, 98–105 (2024).
